# Targeting of ICAM-1 on vascular endothelium under static and shear stress conditions using a liposomal Gd-based MRI contrast agent

**DOI:** 10.1186/1477-3155-10-25

**Published:** 2012-06-20

**Authors:** Leonie EM Paulis, Igor Jacobs, Nynke M van den Akker, Tessa Geelen, Daniel G Molin, Lucas WE Starmans, Klaas Nicolay, Gustav J Strijkers

**Affiliations:** 1 Biomedical NMR, Department of Biomedical Engineering, Eindhoven University of Technology, PO Box 513, 5600 MB, Eindhoven, the Netherlands; 2 Department of Cardiology, Cardiovascular Research Institute Maastricht, Maastricht University, PO Box 616, 6200 MD, Maastricht, the Netherlands; 3 Department of Physiology, Cardiovascular Research Institute Maastricht, Maastricht University, PO Box 616, 6200 MD, Maastricht, the Netherlands

**Keywords:** Molecular MRI, Liposome, ICAM-1, Endothelium, Leukocyte, Shear stress

## Abstract

**Background:**

The upregulation of intercellular adhesion molecule-1 (ICAM-1) on the endothelium of blood vessels in response to pro-inflammatory stimuli is of major importance for the regulation of local inflammation in cardiovascular diseases such as atherosclerosis, myocardial infarction and stroke. *In vivo* molecular imaging of ICAM-1 will improve diagnosis and follow-up of patients by non-invasive monitoring of the progression of inflammation.

**Results:**

A paramagnetic liposomal contrast agent functionalized with anti-ICAM-1 antibodies for multimodal magnetic resonance imaging (MRI) and fluorescence imaging of endothelial ICAM-1 expression is presented. The ICAM-1-targeted liposomes were extensively characterized in terms of size, morphology, relaxivity and the ability for binding to ICAM-1-expressing endothelial cells *in vitro*. ICAM-1-targeted liposomes exhibited strong binding to endothelial cells that depended on both the ICAM-1 expression level and the concentration of liposomes. The liposomes had a high longitudinal and transversal relaxivity, which enabled differentiation between basal and upregulated levels of ICAM-1 expression by MRI. The liposome affinity for ICAM-1 was preserved in the competing presence of leukocytes and under physiological flow conditions.

**Conclusion:**

This liposomal contrast agent displays great potential for *in vivo* MRI of inflammation-related ICAM-1 expression.

## Background

The vascular endothelium plays an essential role in the regulation of the inflammatory phases of atherosclerosis and related cardiovascular complications such as myocardial infarction and stroke [1-3]. In response to local pro-inflammatory stimuli, the endothelial expression of cell adhesion molecules is upregulated to mediate interactions with leukocytes circulating in the blood [4,5]. This allows for leukocyte adhesion to the endothelium, followed by extravasation of leukocytes through the endothelial cell layer to the site of inflammation.

Intercellular adhesion molecule-1 (ICAM-1), a transmembrane immunoglobulin protein that is predominantly expressed on endothelial cells, is of major importance in leukocyte recruitment [6]. Upregulation of ICAM-1 contributes to stable binding of leukocytes and facilitates their transmigration by rearranging the endothelial cytoskeleton and lowering the strength of endothelial cell junctions [7]. Non-invasive *in vivo* molecular imaging of endothelial ICAM-1 expression could therefore provide valuable insights in the progression of cardiovascular disease-related inflammation to improve diagnosis and treatment [8].

Molecular imaging employs sophisticated contrast agents that combine high affinity targeting ligands with imaging labels for *in vivo* visualization of biological processes at the cellular and molecular level. In this study, we introduce a novel liposomal contrast agent functionalized with anti-ICAM-1 (aICAM-1) antibodies for sensitive multimodal magnetic resonance imaging (MRI) and fluorescence imaging of endothelial ICAM-1 expression. MRI enables *in vivo* high-resolution imaging of ICAM-1 expression in an anatomical context, whereas *ex vivo* fluorescence microscopy can be used to study the spatial distribution of the liposomes at the tissue and cellular level [9]. Because of the relatively large diameter of the liposomes (100–150 nm), passive extravasation from the blood is expected to be minimal and liposomes will be largely confined to the blood pool, which facilitates the detection of intravascular targets such as ICAM-1.

The binding of ICAM-1 targeted liposomal contrast agents to endothelial cells was extensively studied *in vitro*. Liposomes were characterized with respect to their size, morphology, longitudinal and transversal relaxivity, and the average number of targeting ligands per liposome was optimized regarding sensitive MRI and fluorescence detection of endothelial ICAM-1 expression. Importantly, this study focused on various aspects that might negatively affect the binding of liposomes to ICAM-1-expressing endothelial cells *in vivo*. In the challenging intravascular environment, ICAM-1 targeted nanoparticles have to compete with circulating leukocytes for binding to ICAM-1 [4,6]. Additionally, blood flow creates endothelial wall shear stress, which shortens the interaction time of nanoparticles with ICAM-1 and imposes hydrodynamic forces on adherent particles, which may result in their detachment [10,11]. Under these conditions, a high binding affinity of ICAM-1-targeted liposomes to the endothelium is crucial to enable *in vivo* imaging of ICAM-1. To address these issues, liposome binding was investigated *in vitro* in the presence of leukocytes and under physiologically relevant shear stress conditions.

## Results

### Liposome characterization

**Figure 1:**
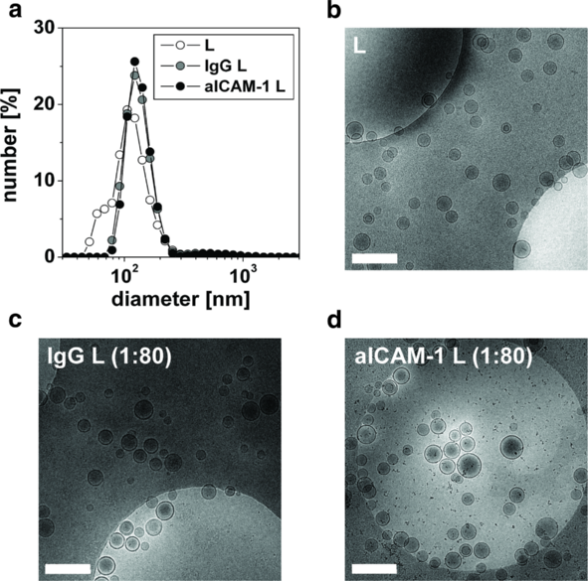
**Characterization of the liposomes by DLS and cryoTEM.(a)** Representative DLS size distributions of L, IgG L (Ab:SATA = 1:80) and aICAM-1 L (Ab:SATA = 1:80) that were prepared from a single batch of liposomes. The three types of liposomes display a similar narrow size distribution. CryoTEM was used to study the morphology of **(b)** L, **(c)** IgG L (Ab:SATA = 1:80) and **(d)** aICAM-1 L (Ab:SATA = 1:80). Lipid suspensions were mainly composed of single unilamellar liposomes. Scale bar = 500 nm.

Antibodies (Ab) were modified using *N*-succinimidyl *S*-acetylthioacetate (SATA) and conjugated to liposomes containing Mal-PEG-DSPE by sulfhydryl-maleimide coupling. Significant binding of antibodies to liposomes was observed for all Ab:SATA ratios used (Table 1). Paramagnetic liposomes were successfully functionalized with murine aICAM-1 or isotype matched non-specific IgG. In Figure 1a representative dynamic light scattering (DLS) spectra are shown of the size distribution of non-functionalized liposomes (L), IgG L and aICAM-1 L, both prepared with antibodies modified with an 80-fold excess of SATA. A single dominant peak was observed for all liposome preparations, indicative of a homogeneous liposome size. Moreover, antibody conjugation did not significantly alter the mean diameter of aICAM-1 L and IgG L compared to L (Table 1).

**Table 1:** Properties of L, IgG L and aICAM-1 L

	**antibody coupling [%]**	**hydrodynamic diameter [nm]**	**r**_**1**_**[mM**^**-1**^ **s**^**-1**^**]**	**r**_**2**_**[mM**^**-1**^ **s**^**-1**^**]**
**NIR-liposomes**				
**L**	2.5 ± 2.8^a,b^	130 ± 6^b^	3.3 ± 0.2^b^	39 ± 1^b^
**IgG L (1:8)**^c^	64 ± 27^d^	140 ± 4	2.9	22
**IgG L (1:20)**	54 ± 18^d^	140 ± 9	2.8	23
**IgG L (1:40)**	89 ± 46^d^	132 ± 10	3.2	46
**IgG L (1:80)**	84 ± 21^d^	134 ± 5	2.7 ± 0.4	38 ± 6
**aICAM-1 L (1:8)**	52 ± 28^d^	137 ± 9	2.9	27
**aICAM-1 L (1:20)**	66 ± 20^d^	133 ± 16	3.3	30
**aICAM-1 L (1:40)**	88 ± 24^d^	156 ± 0^d^	3.1	89
**aICAM-1 L (1:80)**	83 ± 9^d^	141 ± 2	2.7 ± 0.4	53 ± 16
**Rhodamine-liposomes**				
**L**	−0.8 ± 0.8^a^	133 ± 5	4.2 ± 0.8	54 ± 8
**aICAM-1 L (1:80)**	98 ± 4^d^	163 ± 2	3.3 ± 0.1	53 ± 15

Liposomes contained either a NIR or a rhodamine fluorophore. n = 1-5; ^a^ baseline value indicative for the inaccuracy of the protein assay; ^b^ mean ± SEM; ^c^ (1:x) the applied molar ratio of Ab: SATA; ^d^ = p < 0.05 vs. L, ANOVA with LSD correction for NIR-liposomes and *t*-test for rhodamine-liposomes.

To study liposome morphology in higher detail, cryogenic transmission electron microscopy (cryoTEM) was performed (Figure 1b-d). Both non-functionalized and antibody-conjugated liposome suspensions primarily consisted of unilamellar, spherical liposomes, with sizes that were in line with the DLS data.

Paramagnetic liposomes displayed MRI longitudinal and transversal relaxivities of approximately 3.0 mM^-1^ s^-1^ and 20–55 mM^-1^ s^-1^ at 9.4 T, respectively (Table 1). The relaxivities were not significantly affected by liposome functionalization with aICAM-1 or IgG antibodies, though there was a large variation in the observed r_2_ (p > 0.05, aICAM-1 L (1:80) and IgG L (1:80) vs. L).

### Binding of liposomes to ICAM-1

**Figure 2:**
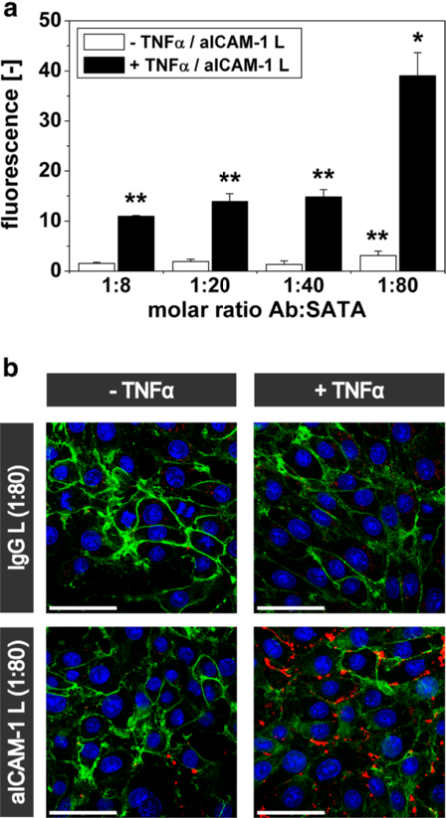
**Binding of aICAM-1 L to non-activated and TNF**α**-activated bEnd.5 cells.(a)** Cellular NIR fluorescence intensity of non-activated and TNFα-activated bEnd.5 cells after 2 h incubation at 37 °C with aICAM-1 L, prepared using different Ab:SATA ratios, quantified with FACS. Data were corrected for cellular fluorescence after incubation with L and reflect the increase in fluorescence by aICAM-1 conjugation. Application of IgG L did not lead to an increase in fluorescence intensity (not shown). * = p < 0.05 vs. all groups, ** = p < 0.05 vs. IgG L, ANOVA with Bonferroni correction. n = 3-4. **(b)** CLSM images illustrating the cellular distribution of aICAM-1 L (Ab:SATA = 1:80) and IgG L (Ab:SATA = 1:80) (red). The cell membrane was labeled with CD31 (green) and cell nuclei were counterstained with DAPI (blue). Laser power 488 nm: 25%, 780 nm: 4%, 633 nm, IgG L: 50%, aICAM-1 L/-TNFα: 10% and aICAM-1 L/+TNFα: 5%. Scale bar = 50 μm.

The ability of aICAM-1 L, prepared using antibodies modified with an 8- to 80-fold excess of SATA, to bind to ICAM-1 was studied using bEnd.5 endothelial cells expressing ICAM-1 at basal (non-activated) or upregulated (TNFα-activated) levels. Non-activated and activated cells incubated with L and various preparations of IgG L had a low NIR-fluorescence intensity (n = 4 per group; not shown). In contrast, binding of aICAM-1 L significantly increased the fluorescence of activated cells up to 100-fold as compared to IgG L. Highest fluorescence intensities, indicative of the highest degree of binding, were observed for liposomes functionalized with aICAM-1 antibodies that were modified with an 80-fold excess of SATA (mean fluorescence intensity = 39.0 ± 4.6, p < 0.05 vs. all groups) (Figure 2a). In addition, the fluorescence of non-activated cells was exclusively enhanced by aICAM-1 L when prepared with an 80-fold excess of SATA (mean fluorescence intensity = 3.1 ± 0.9; p < 0.05 vs. IgG L).

In Figure 2b, representative CLSM images illustrate the cellular distribution of aICAM-1 L and IgG L (Ab:SATA = 1:80). The aICAM-1 L were mainly associated with the cell membrane for both activated and non-activated cells. In accordance with the fluorescence intensity measurements (Figure 2a), abundant binding of aICAM-1 L to activated cells (high ICAM-1 expression) was observed compared to low binding to non-activated cells (basal ICAM-1 expression). Positive secondary labeling of the liposome-associated antibodies with fluorescent goat-anti-rat antibodies revealed that the aICAM-1 moieties on liposomes were available on the outside of the cell membrane, thereby confirming their membrane-bound location (Additional file 1: Figure S1). Liposomal fluorescence was frequently observed in distinct spots (diameter = 2.5 ± 0.3 μm, measurement of 30 spots in 3 images) that were larger than the size of individual liposomes. In contrast, CLSM images showed low intracellular accumulation of IgG L, which could only be detected at high laser power. The cellular distribution of aICAM-1 L and IgG L was independent on the antibody to SATA modification ratio.

Above results showed that aICAM-1 L prepared with an antibody to SATA modification ratio of 1:80 had the highest level of association to ICAM-1 expressing bEnd.5 cells. These liposomes had the unique ability to identify both basal and upregulated levels of ICAM-1 expression (p < 0.05). The liposome formulation with a 1:80 antibody to SATA ratio was therefore used in further experiments described below.

### Liposome concentration-dependence of ICAM-1 binding

**Figure 3:**
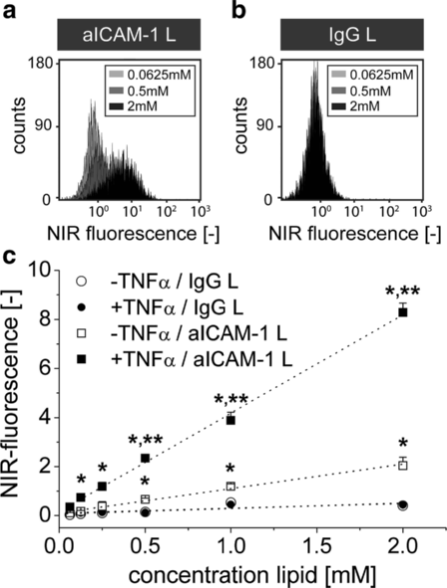
**Binding of aICAM-1 L and IgG L to bEnd.5 cells with varying lipid concentration.** Representative FACS spectra of NIR fluorescence of TNFα-activated bEnd.5 cells after 30 min incubation at 4 °C with **(a)** aICAM-1 L and **(b)** IgG L at different liposome concentrations. **(c)** Mean fluorescence intensity of non-activated and TNFα-activated bEnd.5 cells after incubation with IgG L or aICAM-1 L at liposome concentrations varying from 0.0625-2 mM lipid. * = p < 0.05 vs. IgG L, ** = p < 0.05 vs. aICAM-1 L/-TNFα, *t*-test. n = 3 for aICAM-1 L, n = 1 for IgG L.

The binding of liposomes to endothelial cells as function of the concentration of liposomes in the incubation medium was determined at 4 °C to minimize liposome internalization and ICAM-1 recycling for accurate evaluation of the liposomal ICAM-1 binding interactions. Under these conditions, the NIR-fluorescence intensity of TNFα-activated bEnd.5 cells depended on the concentration of aICAM-1 L in the incubation medium (Figure 3a), but not on the concentration of IgG L (Figure 3b). The mean fluorescence intensity of both activated and non-activated endothelial cells linearly related to the concentration of aICAM-1 L (R^2^ = 0.99), indicating that ICAM-1 binding was not saturated within the concentration range studied (Figure 3c). In contrast, application of IgG L at concentrations up to 2 mM lipid did not result in significant binding to endothelial cells (Figure 3c). Importantly, the binding of aICAM-1 L to activated cells was significantly higher than to non-activated cells (linear slope of 4.0 versus 1.0), proving that the association of aICAM-1 L was also related to the level of ICAM-1 expression.

The minimal aICAM-1 L concentration required to detect upregulated levels of ICAM-1 expression on activated cells using fluorescence activated cell sorting (FACS) was 0.125 mM lipid, whereas 0.5 mM lipid was needed to identify the basal ICAM-1 expression levels on non-activated cells (p < 0.05 aICAM-1 L vs. IgG L). Cellular fluorescence was significantly higher for activated cells as compared to non-activated cells when incubated with aICAM-1 L at lipid concentrations of 0.25 mM and higher.

### MRI detection sensitivity

**Figure 4:**
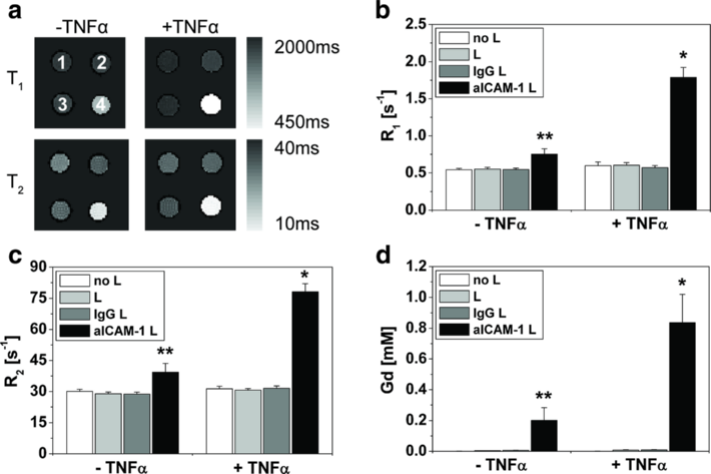
**MRI characterization of bEnd.5 cells incubated with non-targeted and ICAM-1 targeted liposomes.(a)** Representative T_1_ and T_2_ maps of non-activated and TNFα-activated bEnd.5 cells, incubated for 2 h at 37 °C with 1) no liposomes, 2) L, 3) IgG L and 4) aICAM-1 L. Cellular **(b)** R_1_ and **(c)** R_2_ measured at 9.4 T. **(d)** Concentration of gadolinium associated with bEnd.5 cells determined with ICP-MS. * = p < 0.05 vs. all groups, ** = p < 0.05 vs. all non-activated groups, ANOVA with Bonferroni correction. n = 4.

To enable MR-imaging of ICAM-1 expression, paramagnetic aICAM-1 L must specifically and significantly decrease the MR relaxation time parameters. Representative T_1_ and T_2_ maps obtained at 9.4 T (Figure 4a) demonstrated the ability of aICAM-1 L to reduce the relaxation times of cells compared to native cells or those incubated with control liposomes (IgG L or L). This can be recognized from the much brighter color in pellet number 4. From these T_1_ and T_2_ maps, the mean cellular R_1_ and R_2_ were calculated (Figure 4b, c). The R_1_ and R_2_ of TNFα-activated cells were significantly increased by aICAM-1 L (1.8 ± 0.1 s^-1^ and 78 ± 4 s^-1^, respectively) with respect to controls. Importantly, a significant (but smaller) increase in R_1_ and R_2_ was also observed for non-activated cells incubated with aICAM-1 L (0.75 ± 0.07 s^-1^ and 39 ± 4 s^-1^, respectively). Application of control liposomes (IgG L and L) did not alter the cellular R_1_ or R_2_ (p > 0.05). Importantly, aICAM-1 L enabled MRI to distinguish between cells with basal and upregulated levels of ICAM-1 expression (p < 0.05).

Figure 4d illustrates that the increase in the relaxation rates of cells incubated with aICAM-1 L was consistent with a significant association of gadolinium with activated cells (0.84 ± 0.18 mM Gd) and non-activated cells (0.20 ± 0.08 mM Gd) compared to control liposomes. Interestingly, the effective relaxivities r_1_ and r_2_ of cells incubated with aICAM-1 L, which were estimated from the MR-relaxation rates and gadolinium concentrations, were also dependent on the ICAM-1 expression level. The r_1_ improved from 1.2 ± 0.2 mM^-1^ s^-1^ to 1.7 ± 0.5 mM^-1^ s^-1^ for cells with basal and upregulated ICAM-1 expression, respectively, whereas the r_2_ increased from 42 ± 6 mM^-1^ s^-1^ to 103 ± 16 mM^-1^ s^-1^ (p < 0.05). We hypothesize that the increased cellular relaxivity for upregulated levels of ICAM-1 is due to increased immobilization of the liposomes on the cell membrane by steric hindrance.

### Competition from leukocytes

*In vivo* imaging of ICAM-1 expression on inflamed endothelium by aICAM-1 L may be hampered by the presence of circulating leukocytes, which compete with liposomes for binding to ICAM-1. Additionally, leukocytes might phagocytose liposomes, making them unavailable for binding to endothelial ICAM-1. To investigate these interactions *in vitro,* endothelial cells were co-incubated with liposomes and leukocytes that constitutively express CD11b and CD18 (Additional file 2: Figure S2), a receptor pair which binds ICAM-1.

**Figure 5:**
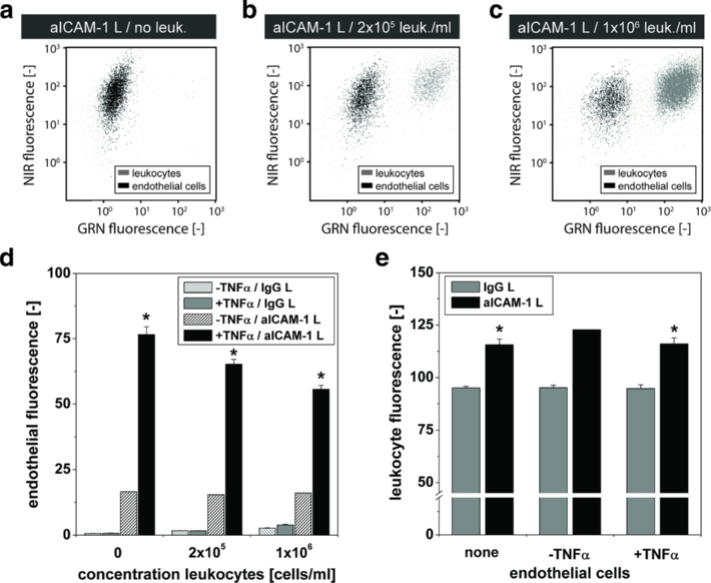
**Binding of aICAM-1 L to bEnd.5 in the competing presence of leukocytes.** Non-activated and TNFα-activated bEnd.5 cells incubated for 2 h at 37 °C with IgG L or aICAM-1 L in the presence of 0, 2.5x10^5^ or 1x10^6^ leukocytes/ml medium. **(a-c)** Typical FACS scatter plots of liposomal NIR-fluorescence vs. GRN (green) fluorescence to distinguish TNFα-activated bEnd.5 cells from RAW cells labeled with calcein (green). **(d)** Endothelial NIR fluorescence intensity. * = p < 0.05 vs. all groups, ANOVA with Bonferroni correction. **(e)** Leukocyte NIR fluorescence intensity. * = p < 0.05 vs. IgG L, ANOVA with Bonferroni correction. n = 3, except for aICAM-1 L/-TNFα n = 1.

The FACS scatter plots in Figure 5a-c reveal that the NIR-fluorescence of TNFα-activated endothelial cells incubated with aICAM-1 L slightly decreased with increasing concentration of leukocytes. More extensively, in Figure 5d the mean fluorescence intensity originating from aICAM-1 L and IgG L bound to either activated or non-activated endothelial cells is shown for increasing leukocyte concentrations. A moderate, but significant decline in endothelial fluorescence from 76.5 ± 3.0 (no leukocytes) to 65.2 ± 1.9 (2x10^5^ leukocytes/ml) and 55.6 ± 1.5 (1x10^6^ leukocytes/ml) was observed for aICAM-1 L, indicating that leukocytes indeed reduced the association of aICAM-1 L with activated endothelium. Nevertheless, the fluorescence of activated endothelial cells was strongly enhanced by aICAM-1 L compared to IgG L, regardless of the presence of leukocytes (p < 0.05). The ability of aICAM-1 L to bind to non-activated endothelial cells was not compromised by leukocytes, as the endothelial fluorescence was independent on the concentration of leukocytes in this case (Figure 5d).

Importantly, leukocytes exhibited massive accumulation of IgG L and aICAM-1 L, as illustrated in Figure 5e, in which the mean leukocyte fluorescence intensity is shown. Upon incubation with IgG L, the fluorescence of leukocytes was increased 135-fold compared to endothelial cells (compare Figure 5d and Figure 5e). Additionally, leukocyte fluorescence was significantly higher when cells were incubated with aICAM-1 L (116 ± 3) compared to IgG L (95.0 ± 0.8), which is in accordance with the expression of ICAM-1 by leukocytes (Additional file 2: Figure S2). The association of liposomes with leukocytes was independent of the presence of endothelial cells, both for non-activated and activated endothelial cells.

### Liposomal ICAM-1 binding under shear stress conditions

**Figure 6:**
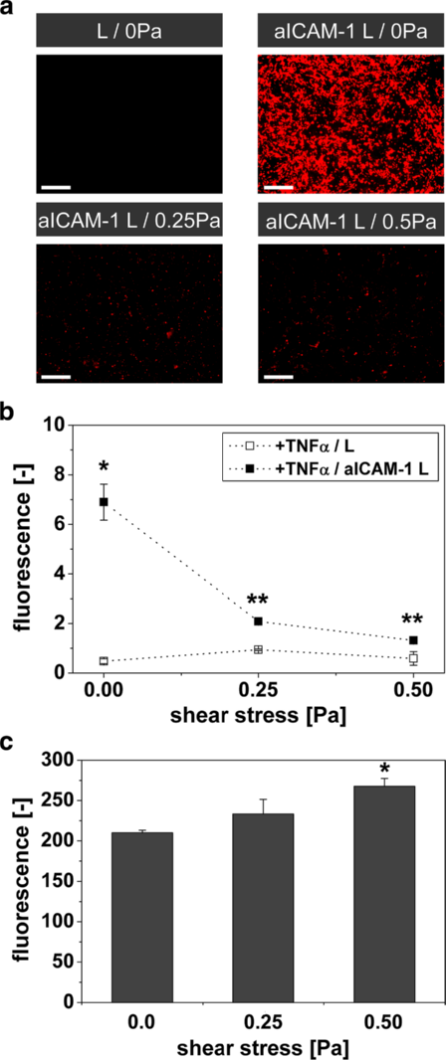
**Binding of aICAM-1 L to bEnd.5 in the competing presence of shear stress.** TNFα-activated cells incubated with L or aICAM-1 L for 2 h at 37 °C and shear stress values of 0, 0.25 or 0.5 Pa. **(a)** Fluorescence microscopy of rhodamine lipids in liposomes adherent to bEnd.5 cells (red). Scale bar = 100 μm. **(b)** Cellular rhodamine fluorescence intensity quantified with FACS. * = p < 0.05 vs. all groups, ANOVA with Bonferroni correction, ** = p < 0.05 vs. IgG L, *t*-test. n = 2-5. **(c)** ICAM-1 expression at different shear stress levels. * = p < 0.05 vs. 0 Pa, ANOVA with Bonferroni correction. n = 3.

The *in vivo* binding of aICAM-1 L to vascular endothelium requires fast and strong interactions with ICAM-1 to resist the continuous shear stress generated by blood flow. Therefore, the binding potential of aICAM-1 L was studied under physiologically relevant wall shear stress values up to 0.5 Pa *in vitro*[12]. Fluorescence microscopy images of TNFα-activated endothelial cells incubated with L (0 Pa) and aICAM-1 L (0, 0.25 and 0.5 Pa) are shown in Figure 6a. Fluorescence originating from binding of aICAM-1 L was detected at all applied shear stress values, whereas no significant fluorescence was observed after application of L. However, shear stress elevation resulted in a reduction of the fluorescence of aICAM-1 L, indicative of decreased binding to ICAM-1, compared to static conditions.

After harvesting the cells from the flow chamber, the fluorescence intensity was quantified by FACS (Figure 6b). The application of flow reduced the ability of aICAM-1 L to adhere to ICAM-1 on endothelial cells, as evidenced by a significant decrease in cellular fluorescence from 6.9 ± 0.7 (0 Pa) to 2.1 ± 0.1 (0.25 Pa) and 1.3 ± 0.1 (0.5 Pa). A confounding factor could be that wall shear stress altered the ICAM-1 expression levels. Fluorescent evaluation of ICAM-1 expression showed that ICAM-1 expression levels were increased at shear stress levels of 0.25 Pa and 0.5 Pa (Figure 6c), in agreement with previous findings [13,14]. Therefore, the reduction of nanoparticle binding under flow conditions is somewhat higher than the numbers indicate. Nevertheless, the binding of aICAM-1 L to endothelial cells remained significantly higher than of L at both shear stress levels.

## Discussion

Excessive recruitment of leukocytes to sites of atherosclerosis, myocardial infarction or stroke is implicated in adverse disease progression [15,16]. Clinical treatment decision-making might therefore substantially benefit from *in vivo* imaging readouts of the local inflammatory status. The *in vivo* MR-imaging of cell adhesion molecules expressed on inflamed vascular endothelium is of particular interest, considering their crucial role in mediating leukocyte extravasation [4]. Previous studies have demonstrated that targeted iron-oxide-based MR-contrast agents are able to create hypo-intense signals on T_2_^*^-weighted images in regions of VCAM-1 or P-selectin expression in mouse models of atherosclerosis and brain inflammation [17-19]. Alternatively, the use of targeted Gd-based probes, which create signal hyperenhancement on T_1_-weighted images, has been explored as well [20-22]. Choi et al. used anti-ICAM-1 antibodies decorated with Gd-DTPA moieties to highlight muscular inflammation on *in vivo* MRI, whereas Sipkins et al. performed *ex vivo* MRI to visualize brain inflammation using paramagnetic liposomes [20,21]. In this study, a novel paramagnetic ICAM-1-targeted liposomal contrast agent was designed and, importantly, its interaction with ICAM-1 was studied *in vitro* under conditions that mimic the challenging environment encountered in the circulation.

Liposomes containing Gd-DOTA-DSPE previously have been characterized with respect to their MRI properties at 1.4 T, which is close to a clinical field strength of 1.5 T [23]. At 1.4 T, Gd-DOTA-DSPE liposomes have a high longitudinal relaxivity compared to frequently used Gd-DTPA-BSA liposomes, which is caused by improved water access to Gd by the use of a small linker between DSPE and DOTA as well as by the different exchange dynamics of water bound to the Gd ion that is much faster for the tetra-carboxylate DOTA ligand than for the bis-carboxoamide linear DTPA ligand [23,24]. A high longitudinal relaxivity will facilitate sensitive detection of the Gd-DOTA-DSPE liposomes. At the high preclinical field strength used in this study (9.4 T) one cannot fully exploit this high longitudinal relaxivity. Nevertheless, relaxivity numbers are expressed in terms of relaxivity per Gd atom and the very high payload of Gd (~10^5^ Gd per liposome) results in much higher relaxivity values per nanoparticle and thus sensitive detection on T_1_-weighted images [25]. A possible drawback of the use of these liposomes is the enhanced r_2_/r_1_ ratio at 9.4 T, which might introduce a considerable T_2_-weighting in the images. However, by carefully choosing the MR-imaging parameters, T_2_ effects can be minimized to make optimal use of the T_1_-lowering properties of liposomes.

The binding of liposomes to ICAM-1 expressing endothelial cells was optimized by improving the coupling of aICAM-1 antibodies by increasing the extent of antibody thiolation. The antibody-coupling efficacy was enhanced by 30-60% using an 80-fold excess of SATA compared to an 8-fold excess. The 1:80 antibody to SATA modification ratio resulted in a liposomal antibody density of 960–1200 Ab/μm^2^, which compares well to recent studies using ICAM-1 targeted polystyrene beads or ultrasound microbubbles [11,26,27]. The ability of aICAM-1 L to adhere to ICAM-1 expressing endothelial cells was not compromised by the extensive antibody thiolation (Figure 2a). Furthermore, the association of aICAM-1 L with endothelial ICAM-1 varied linearly with the concentration of liposomes (Figure 3c) and did not saturate within a physiologically relevant range (up to 2 mM lipid). This agrees with earlier studies using fluorescent liposomes where ICAM-1 binding was only saturated at higher liposome concentrations (5 to 12 mM lipid) [28,29]. Importantly, the binding of aICAM-1 L was dependent on the level of ICAM-1 expression on the endothelial cell membrane (Figures 2a, 3c), which is encouraging for the use of aICAM-1 L for quantitative *in vivo* MR-imaging of ICAM-1.

The sensitivity of MRI at 9.4 T was sufficient to differentiate between basal and upregulated levels of ICAM-1 expression *in vitro* based on the MR relaxation rates of endothelial cells after binding of paramagnetic aICAM-1 L (Figure 4b, c). Interestingly, the contribution of aICAM-1 L to the cellular R_1_ was larger than to the R_2_, despite the high r_2_/r_1_ ratio of liposomes in buffer at 9.4 T. This pronounced effect of aICAM-1 L on endothelial cell R_1_ will facilitate the detection of ICAM-1 expression on vascular endothelium *in vivo* with T_1_-weighted MRI. Additionally, this finding indicated that the longitudinal relaxivity of aICAM-1 L could be fully exploited and was not restricted by limited water exchange as a result of internalization and subsequent compartmentalization of liposomes into endosomes, as was previously observed for α_v_β_3_-targeted liposomes [30,31]. CLSM indeed showed that aICAM-1 L were not internalized, but were mainly bound to the extracellular side of the cell membrane (Additional file 1: Figure S1) – a distinct advantage to other ICAM-1 specific nanoparticles [29,32,33]. Previously, though, Mastrobattista et al. have shown internalization of ICAM-1 targeted liposomes by human lung epithelial cells, indicating that the human ICAM-1 receptor is able to internalize liposomes [29]. Moreover, Muro et al. have performed extensive studies on the internalization pathway of ICAM-1 targeted nanometer and micrometer sized fluorescent particles to clarify the exact mechanism for ICAM-1 mediated internalization in endothelial cells [32-34]. The observed lack of internalization of aICAM-1 L in our study could also be related to differences in the condition of bEnd.5 endothelial cells compared to human umbilical vein or lung epithelial cells used in other studies, thereby resulting in an inability to internalize aICAM-1 L through cell-adhesion-molecule-mediated endocytosis.

*In vitro* competition experiments showed that the association of aICAM-1 L with ICAM-1 on endothelial cells was lowered in the presence of leukocytes (Figure 5d). This is probably related to occupation and steric hindrance of ICAM-1 receptors by the CD11b/CD18-expressing leukocytes. *In vivo,* partial blocking of ICAM-1 receptors on inflamed endothelium by leukocytes could result in an underestimation of the ICAM-1 expression level by aICAM-1 L. Moreover, both aICAM-1 L and IgG L were internalized by phagocytotic leukocytes *in vitro* (Figure 5e). This might cause false positive non-specific MR-signal enhancement *in vivo* by extravasation of liposome-laden leukocytes at sites of inflammation. Nevertheless, in the circulation quiescent leukocytes require activation by local inflammatory stimuli and therefore blood-pool accumulation of liposomes in leukocytes is expected to be low.

The capacity of aICAM-1 L to bind to endothelial cells lining the vasculature could be affected by blood flow, which reduces the interaction time for liposome binding and imposes torque and shear forces on the adherent liposomes, as was previously observed for targeted fluorescent microbeads and ultrasound microbubbles [10,11,34-36]. In this study, *in vitro* binding of aICAM-1 L to ICAM-1 was reduced with increasing shear stress within a physiologically relevant range (Figure 6b) [12]. Nevertheless, the ability of aICAM-1 L to bind to endothelial cells in the microcirculation *in vivo* might be improved by the effect that erythrocytes preferentially occupy the center of blood vessels, thereby increasing the effective liposome concentration near the vessel wall and enhancing their probability to interact with the endothelium [37]. Furthermore, there are several options to improve the binding of aICAM-1 L to endothelial cells. Recently, Calderon et al. observed improved interaction kinetics and total bond strength of ICAM-1-targeted microbeads with endothelial cells, when increasing the nanoparticle’s antibody density from 1100 Ab/μm^2^ to 4100 Ab/μm^2^[11]. This is probably related to an increased multivalency of nanoparticle-endothelial cell interactions. Furthermore, incorporation of PEG-polymers in the liposome bilayer, as used in our liposome formulation, may have a positive effect on the aICAM-1/ICAM-1 interaction strength. PEG is capable of enhancing the bond lifetime between ligands on nanoparticles and their corresponding receptors on cells under hydrodynamic conditions by increasing the bond flexibility [38]. Moreover, liposomes might be functionalized with more than one type of targeting ligand to optimize endothelial cell association under flow conditions, as previously shown for VCAM-1 and P-selectin targeted microbubbles by Ferrante et al. [26,39].

## Conclusions

In this study, a high-relaxivity ICAM-1-binding liposomal MR-contrast agent was developed that 1) showed strong binding to endothelial cells that depended on both the ICAM-1 expression level and the concentration of liposomes, 2) could distinguish between basal and upregulated levels of ICAM-1 expression by MRI and 3) displayed significant binding to endothelial ICAM-1 even in the competing presence of leukocytes and under physiological flow conditions. Taken together, the ability of ICAM-1 targeted liposomes to bind ICAM-1 under these harsh conditions might allow this contrast agent to visualize ICAM-1 in a variety of cardiovascular and neurological diseases using *in vivo* MR-imaging.

## Methods

### Liposome preparation

Liposomes were composed of 1,2-distearoyl-*sn*-glycero-3-phosphocholine (DSPC, Lipoid, Steinhausen, Switzerland), cholesterol (Avanti Polar Lipids, Alabaster, USA), gadolinium-DOTA-1,2-distearoyl-*sn*-glycero-3-phosphoethanolamine (Gd-DOTA-DSPE, SyMO-Chem, Eindhoven, the Netherlands), DSPE-*N*-[methoxy(poly(ethyleneglycol))2000] (PEG-DSPE, Lipoid), DSPE-*N*-[maleimide\(poly(ethyleneglycol))2000] (Mal-PEG-DSPE, Avanti Polar Lipids) and near-infrared664-DSPE (NIR664-DSPE, SyMO-Chem) or 1,2-dipalmitoyl-*sn*-glycero-3-phosphoethanolamine-*N*-(lissamine rhodamine B sulfonyl) (rhodamine-PE, Avanti Polar Lipids) in a molar ratio of 1.1:1:0.75:0.075:0.075:0.003. Lipid films were prepared by rotary evaporation (30 °C) of 50 μmol lipid dissolved in chloroform and methanol (8:1 v/v), with additional drying under N_2_. To obtain liposomes, lipid films were hydrated at 65 °C for 10 min in 8 ml HEPES-buffered saline (HBS, pH 6.7), composed of 10 mM HEPES and 135 mM NaCl, followed by extrusion at 65 °C through polycarbonate membrane filters of 400 nm (2x) and 200 nm (10x) [23].

### Liposome functionalization with antibodies

Monoclonal mouse aICAM-1 and isotype-matched control IgG antibodies (clone YN1/1.7.4 and RTK4530, BioLegend, Uithoorn, the Netherlands) were covalently coupled to Mal-PEG-DSPE through thioether linkage to obtain aICAM-1 liposomes (aICAM-1 L) and IgG liposomes (IgG L), respectively [40]. For this purpose, acetylthioacetate moieties were introduced on antibodies by modification with *N*-succinimidyl *S*-acetylthioacetate (SATA, Sigma-Aldrich, Zwijndrecht, the Netherlands) for 40 min at room temperature (RT). Various molar ratios of Ab:SATA (1:8, 1:20, 1:40 and 1:80) were tested to optimize antibody coupling to liposomes. Free SATA was removed by washing in HBS (pH 6.7) on a Vivaspin concentrator (30 kDa cut-off) by centrifugation at 3000 g and 4 °C. Acetylthioacetate groups were converted into free thiols by deacetylation with hydroxylamine (pH 7.0) for 1 h (RT). Directly thereafter, antibodies and liposomes were mixed at 50 μg protein/μmol lipid at 4 °C under N_2_. Coupling of the antibodies to liposomes continued overnight, after which the liposomes were diluted in HBS (pH 7.4). Liposomes were separated from non-conjugated antibodies by ultracentrifugation (55,000 rpm; 45 min; 4 °C). Liposomes were resuspended in HBS (pH 7.4) to a final concentration of 50–70 mM lipid and stored at 4 °C until further use.

### Liposome characterization

Liposomal phospholipid concentration was quantified with a phosphate determination according to Rouser [41]. Antibody coupling efficacy to liposomes was determined by a Lowry-based protein assay (Bio-Rad, Veenendaal, the Netherlands), corrected for the presence of lipids [42]. The average hydrodynamic number-weighted diameter and size distribution of the liposomes were estimated by DLS of a 633 nm laser on a Zetasizer Nano S (Malvern Instruments, Worcestershire, UK) at RT.

Liposome morphology was evaluated with cryoTEM. Samples were vitrified on carbon-coated cryoTEM grids with a vitrification robot (Vitrobot Mark III, FEI, Hillsboro, USA). Imaging was performed on a Tecnai 20 Sphera TEM instrument (FEI) equipped with a LaB6 filament (200 kV) and Gatan cryoholder (approximately −170 °C) at 6500x magnification.

Liposomal longitudinal and transversal relaxation times (T_1_ and T_2_) were determined with a 9.4 T horizontal bore scanner (Bruker BioSpin GmbH, Ettlingen, Germany) using a 35-mm-diameter quadrature RF-coil (Rapid Biomedical, Rimpar, Germany). T_1_ was obtained with an inversion-recovery segmented FLASH sequence with TR = 15 s and TI = 72.5-4792.5 ms (60 inversion times). For T_2_ measurements, a spin echo sequence was used with TR = 2 s and TE = 9-288 ms (32 echoes). Quantitative T_1_ and T_2_ values were obtained by fitting the MR-data with mono-exponential relaxation curves in Mathematica 6 (Wolfram Research Europe, Oxfordshire, UK). Relaxivities (r_1_ and r_2_ in mM^-1^ s^-1^) were determined from R_i_ = R_i,0_ + r_i·_[Gd], with i ∈ {1,2}, R_i_ = 1/T_i_, R_i,0_ = R_i_ of sample without liposomes and [Gd] varying from 0.01-1 mM Gd. Relaxivities are expressed in terms of Gd concentration rather than nanoparticle concentration.

### Cell culture

Mouse brain endothelioma cells, bEnd.5 (European Collection of Animal Cell Cultures (ECACC)), were cultured in low glucose DMEM, supplemented with 10% fetal bovine serum (FBS) and 5 μM 2-mercaptoethanol. The bEnd.5 cells display a basal expression of ICAM-1. ICAM-1 expression was upregulated by 24 h activation with 40 ng/ml recombinant tumor necrosis factor-α (TNFα, PeproTech EC Ltd., London, UK) as shown in Additional file 3: Figure S3. Mouse leukocytes, RAW 264.7 (ECACC), were maintained in RPMI medium, containing 10% FBS, 2 mM L-glutamine and 10^5^ U/l penicillin/streptomycin. Prior to experiments, RAW cells were fluorescently labeled with 1 μM calcein AM (Invitrogen, Bleiswijk, the Netherlands) for 30 min (37 °C). Excess calcein was removed by centrifugation (2x5 min, 500 g).

### Liposomal ICAM-1 binding under static conditions

The ability of aICAM-1 L to specifically bind to non-activated and TNFα-activated bEnd.5 cells was first investigated under static incubation conditions. To identify the liposome formulation with highest level of ICAM-1 binding, cells were incubated for 2 h at 37 °C with aICAM-1 L or IgG L, prepared with various ratios of Ab:SATA, or non-functionalized liposomes (L) at a concentration of 1 mM lipid. Afterwards, cells were washed with medium and phosphate buffered saline (PBS) to remove non-bound liposomes. For fluorescence intensity quantification, cells were harvested with trypsin/EDTA, fixed in 4% paraformaldehyde (PFA) and stored in 0.01% sodium-azide, whereas for confocal laser scanning microscopy (CLSM) cells cultured in microscopy chambers (Ibidi GmbH, München, Germany) were fixed in 4% PFA and stored in PBS. Prior to CLSM, cell membranes were labeled with biotin rat anti-mouse CD31 (10 μg/ml, BioLegend) conjugated to streptavidin-fluorescein isothiocyanate (FITC) (5 μg/ml, BioLegend). Cell nuclei were labeled with 0.1 μg/ml 4’6-diamidino-2-phenylindole dihydrochloride (DAPI, Invitrogen). In separate samples, goat anti-rat Alexa488 (10 μg/ml, Invitrogen) was added to visualize extracellularly located antibody-conjugated liposomes.

The relation between the liposome concentration in the incubation medium and the extent of liposome binding to ICAM-1 was studied at 4 °C to inhibit internalization of ICAM-1-liposome complexes. Non-activated and TNFα-activated cells were incubated for 30 min at 4 °C with various concentrations of aICAM-1 L or IgG L (0.0625-2 mM lipid). Non-bound liposomes were removed by washing and cells were processed for fluorescence intensity quantification as described above.

To determine the sensitivity of MRI to detect basal and upregulated levels of ICAM-1 expression, non-activated and TNFα-activated cells were incubated for 2 h at 37 °C with aICAM-1 L, IgG L or L (1 mM lipid). Next, cells were washed, harvested and fixed in 4% PFA. A loosely packed cell pellet was allowed to form at 4 °C.

### Competition from leukocytes

The binding of liposomes to endothelial cells was also evaluated in the competing presence of leukocytes by co-incubation of non-activated or TNFα-activated bEnd.5 cells (2 h at 37 °C) with 0, 2x10^5^ or 1x10^6^ calcein-labeled RAW cells/ml and aICAM-1 L or IgG L (1 mM lipid). To study direct interactions of leukocytes with liposomes, RAW cells were incubated for 2 h at 37 °C with aICAM-1 L or IgG L (1 mM lipid) in the absence of bEnd.5 cells. After incubation, cells were washed and samples were prepared for fluorescence intensity quantification. To determine ICAM-1, CD11b and CD18 expression levels on RAW cells, cells were labeled by rat anti-mouse antibodies against ICAM-1 (10 μg/ml) conjugated to goat anti-rat Cy3 (5 μg/ml), biotin CD11b (10 μg/ml) in combination with streptavidin-Cy3 (5 μg/ml) or CD18 R-phycoerythrin (PE) (4 μg/ml) (all antibodies from BioLegend).

### Liposomal ICAM-1 binding under shear stress conditions

The effect of shear stress on the ability of aICAM-1 L to associate with endothelial ICAM-1 was studied with a unidirectional flow system (Ibidi GmbH) [43]. The system was calibrated at a shear stress of 0.25 Pa and 0.5 Pa, taking into account the viscosity of medium containing liposomes (η = 0.75 mPa·s at both shear stress values). The bEnd.5 cells were cultured and activated with TNFα on flow chamber microscopy slides (50x5x0.8 mm^3^ μ-slide, Ibidi GmbH) under static conditions. Subsequently, TNFα-activated bEnd.5 cells were incubated with aICAM-1 L or L (1 mM lipid) at a constant shear stress of 0, 0.25 or 0.5 Pa in closed flow chambers at 37 °C. After 2 h, cells were washed and the rhodamine fluorescence of adherent liposomes was evaluated with a Leica DMI 3000B microscope (Leica Microsystems, Rijswijk, Netherlands) equipped with a Leica EL6000 light source and 590 nm long pass filter. Afterwards, samples were prepared for fluorescence intensity quantification. To evaluate ICAM-1 expression at different flow rates, cells were labeled with rat anti-mouse ICAM-1 (20 μg/ml) and goat anti-rat Alexa488 (40 μg/ml).

### Cellular fluorescence intensity

The fluorescence intensity of cell-associated rhodamine- or NIR664-lipids and fluorescently labeled antibodies was quantified by FACS on a Guava EasyCyte 8HT (Millipore, Billerica, USA). NIR664 was excited with a 640 nm laser and detected using a 661/19 nm band pass (BP) filter. Rhodamine, Cy3 and PE were detected with a 488 nm laser combined with a 583/26 nm BP filter, whereas calcein-labeled RAW cells and Alexa488 were captured with a 525/30 BP filter. Mean cellular fluorescence intensity was determined with Kaluza 1.0 software (Beckman Coulter) and was corrected for cellular autofluorescence, unless mentioned otherwise.

### Cellular distribution of liposomes

The cellular location of the liposomes was studied with CLSM. NIR664-lipids were visualized with an LSM 510 META system (Carl Zeiss B.V., Sliedrecht, Netherlands) equipped with a 633 nm HeNe laser (5.0 mW) in combination with a 680/60 nm BP filter. Cell membranes and extracellular liposomes labeled with Alexa488 were detected with a 488 nm argon laser (13.5 mW) using a 525/50 nm BP filter. A Ti:Sapphire laser tuned to 780 nm (2925.0 mW) was used for two-photon excitation of DAPI, whose fluorescence was captured with a 460/50 BP filter. All images were acquired with a 63x oil immersion objective at 0.07x0.07 μm^2^ in-plane resolution (2048x2048 matrix, 4 averages).

### Cellular relaxation rates and relaxivities

Cellular relaxation rates (R_1_ and R_2_) were determined at 9.4 T as described above. Cellular relaxivities (r_1_ and r_2_) were calculated according to R_i_ = R_i,0_ + r_i·_[Gd], with i ∈ {1,2}, R_i,0_ = R_i_ of cells incubated without liposomes. To quantify gadolinium concentrations, the volume of the cell pellets was obtained from 3D FLASH MR-images (9.4 T; TR = 25 ms; TE = 3.7 ms; flip angle = 30^o^; 100 μm^3^ isotropic resolution) with Osirix Software ( http://www.osirix-viewer.com), whereas the gadolinium content was quantified by inductively coupled plasma mass spectrometry (ICP-MS) using a DRCII (Perkin Elmer, Waltham, USA) after destruction in 1:2 (v/v) nitric acid and perchloric acid at 180 °C.

### Statistics

One-way analysis of variance (ANOVA) with Bonferroni correction for multiple group comparisons and ANOVA with LSD correction or Student’s *t*-test for comparison between two groups were used to test for significant differences (p < 0.05). Data were presented as mean ± SEM.

## Abbreviations

Ab: Antibody; CLSM: Confocal laser scanning microscopy; cryoTEM: Cryogenic transmission electron microscopy; DLS: Dynamic light scattering; FACS: Fluorescence activated cell sorting; ICAM-1: Intercellular adhesion molecule-1; aICAM-1: Anti-ICAM-1; ICP-MS: Inductively coupled plasma mass spectrometry; aICAM-1 L: aICAM-1 liposomes; IgG L: IgG liposomes; MRI: Magnetic resonance imaging; SATA: N-succinimidyl S-acetylthioacetate.

## Competing interests

The authors declare that they have no competing interests.

## Authors’ contributions

All authors added intellectual content, read and approved the final version. LP: Designed the study, performed experiments, performed data analysis, performed statistical analysis, prepared and edited the manuscript. IJ, NvdA, TG, LS: Performed experiments, performed data analysis, edited the manuscript. DM: Edited the manuscript. KN: Co-designed the study, edited the manuscript. GS: Principal investigator, designed the study, prepared and edited the manuscript. All authors read and approved the final manuscript.

## Supplementary Material

Additional file 1 **Figure S1.** CLSM images of activated bEnd.5 cells incubated with aICAM-1 L (Ab:SATA = 1:80). (left) NIR fluorescence from the liposomes in red. (middle) In green, fluorescence from extracellularly located antibodies labeled with goat anti-rat Alexa488. (right) Merged image shows the co-localization (yellow) of aICAM-1 L (red) and Alexa488- IgG (green), thereby confirming the extracellular location of aICAM-1 L. Laser power 488 nm: 3%, 633 nm: 5%. Scale bar = 50 μm. Click here for file

Additional file 2 **Figure S2.** ICAM-1, CD11b and CD18 expression levels on RAW cells quantified with FACS. Fluorescence intensities were corrected for non-specific binding of goat anti-rat Cy3. n = 1. Click here for file

Additional file 3 **Figure S3.** ICAM-1 and CD31 expression levels on non-activated (−TNFα) and activated (+TNFα) bEnd.5 cells quantified with FACS. Fluorescence intensities were corrected for non-specific binding of goat anti-rat Alexa488. The fluorescence of non-activated cells labeled with aICAM-1 antibodies did not exceed the fluorescence of cells incubated with goat anti-rat Alexa488 only. n = 3 for ICAM-1 and n = 1 for CD31. * = p < 0.05 vs. –TNFα/ICAM-1, *t*-test. Click here for file
