# Supplementary figures and images for: Targeting of ICAM-1 on vascular endothelium under static and shear stress conditions using a liposomal Gd-based MRI contrast agent
Source: J Nanobiotechnology. 2012 Jun 20;10:25. doi: 10.1186/1477-3155-10-25 (PMC3563567; doi:10.1186/1477-3155-10-25)

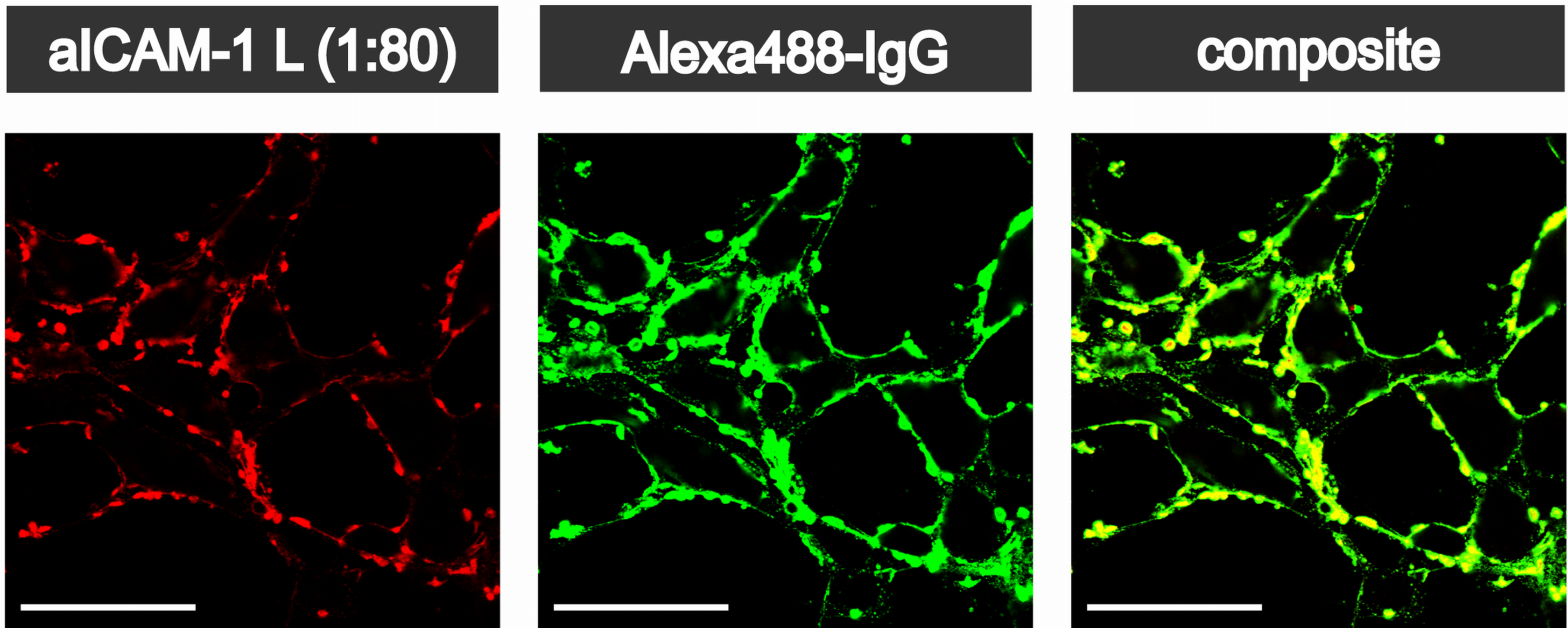

Supplement: Additional file 1 — Figure S1. CLSM images of activated bEnd.5 cells incubated with aICAM-1 L (Ab:SATA = 1:80). (left) NIR fluorescence from the liposomes in red. (middle) In green, fluorescence from extracellularly located antibodies labeled with goat anti-rat Alexa488. (right) Merged image shows the co-localization (yellow) of aICAM-1 L (red) and Alexa488- IgG (green), thereby confirming the extracellular location of aICAM-1 L. Laser power 488 nm: 3%, 633 nm: 5%. Scale bar = 50 μm. [file 1477-3155-10-25-S1.tiff]

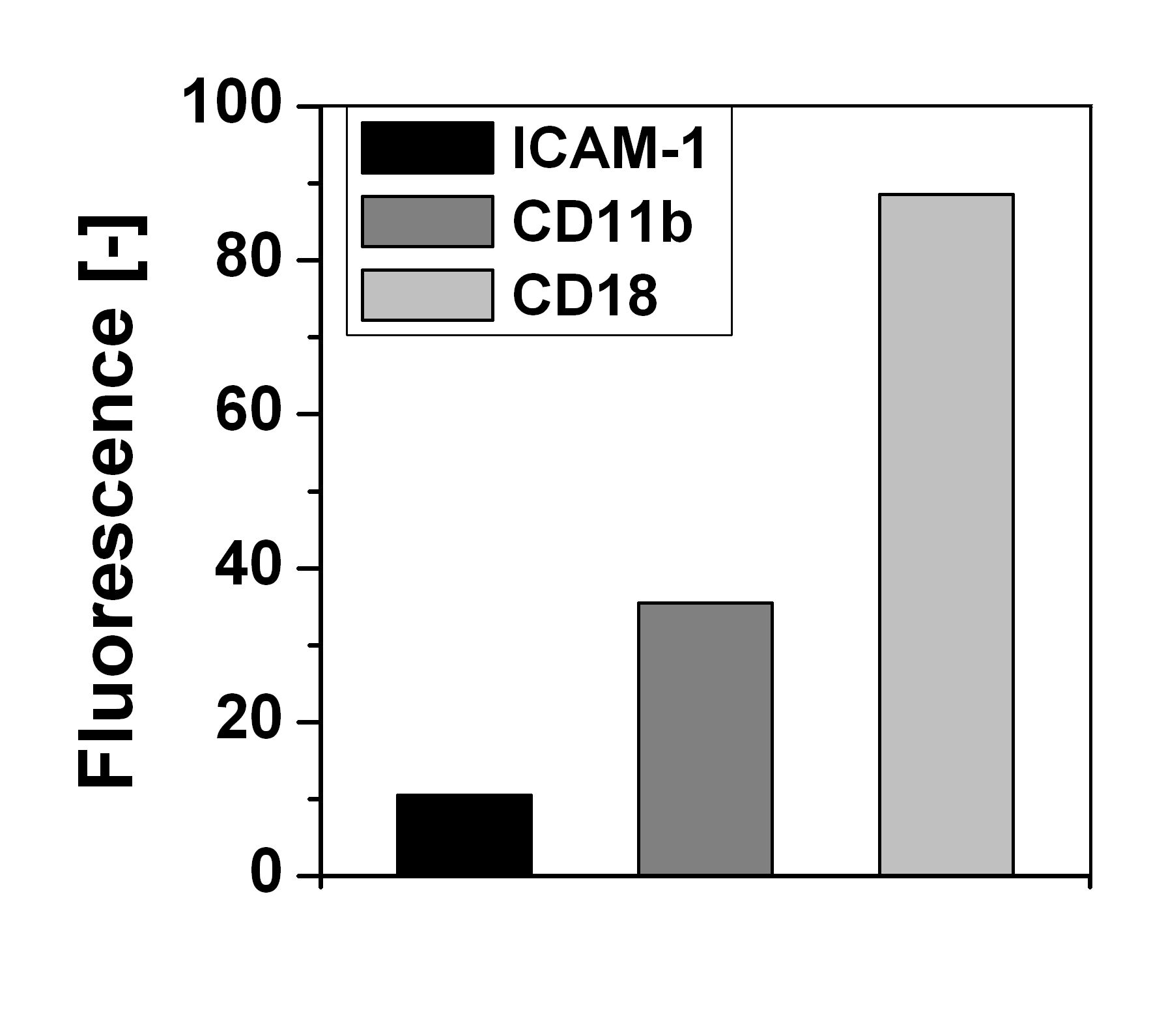

Supplement: Additional file 2 — Figure S2. ICAM-1, CD11b and CD18 expression levels on RAW cells quantified with FACS. Fluorescence intensities were corrected for non-specific binding of goat anti-rat Cy3. n = 1. [file 1477-3155-10-25-S2.tiff]

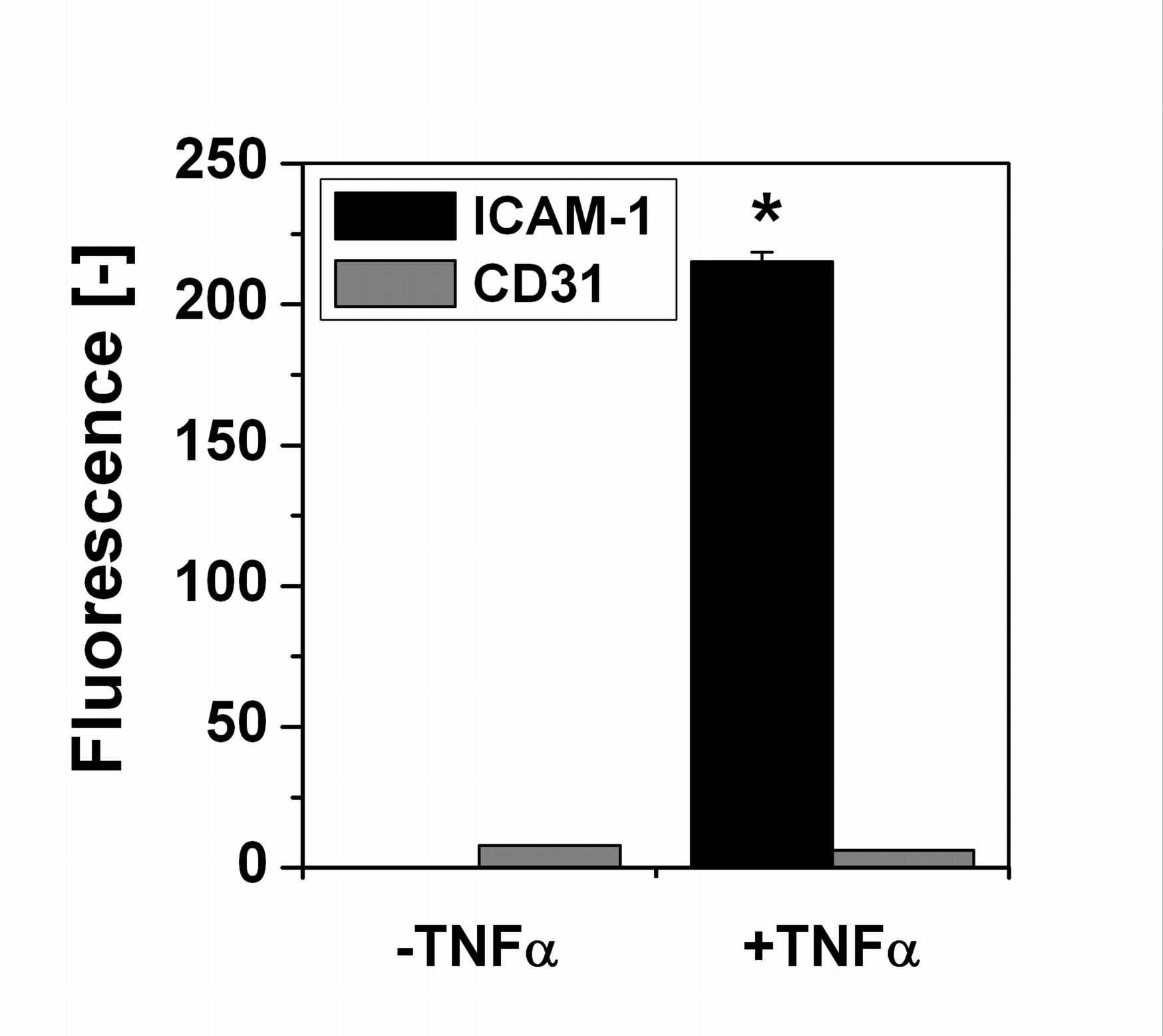

Supplement: Additional file 3 — Figure S3. ICAM-1 and CD31 expression levels on non-activated (−TNFα) and activated (+TNFα) bEnd.5 cells quantified with FACS. Fluorescence intensities were corrected for non-specific binding of goat anti-rat Alexa488. The fluorescence of non-activated cells labeled with aICAM-1 antibodies did not exceed the fluorescence of cells incubated with goat anti-rat Alexa488 only. n = 3 for ICAM-1 and n = 1 for CD31. * = p < 0.05 vs. –TNFα/ICAM-1, t-test. [file 1477-3155-10-25-S3.tiff]
